# Seroprevalence of rubella virus infection among antenatal care clients of Halaba Town public health facilities, southern Ethiopia

**DOI:** 10.1038/s41598-023-34444-w

**Published:** 2023-05-03

**Authors:** Bedilu Asrat, Techalew Shimelis, Abiyu Ayalew Assefa, Siraj Hussen

**Affiliations:** 1 Southern Nations, Nationalities, and Peoples’ Region Public Health Institute, Hawassa, Ethiopia; 2 School of Medical Laboratory Sciences, College of Medicine and Health Sciences, Hawassa University, Hawassa, Ethiopia; 3 Department of Public Health, Hawassa College of Health Sciences, Hawassa, Ethiopia

**Keywords:** Microbiology, Diseases, Risk factors

## Abstract

Rubella virus infection during pregnancy has several effects on the developing fetus. However, little is known about the epidemiology of the infection in Ethiopia. A cross-sectional study was conducted to assess the seroprevalence of rubella virus infection on consecutive 299 pregnant women attending antenatal care clinics in public health facilities in Halaba Town, Southern Ethiopia. Structured questionnaires were used to collect information on socio-demographic and reproductive characteristics. Venous blood samples were collected, and sera were tested for anti-rubella IgM and IgG using the enzyme-linked immunosorbent assay. Anti-rubella IgG and IgM were detected in 265 (88.6%) and 15 (5.0%) of 299 participants, respectively. Pregnant women in their first trimester [crude odds ratio (cOR) = 4.26; 95% CI (1.47, 12.4)] were at increased risk of having anti-rubella IgM compared to those in their second and third trimesters. Urban residents [cOR = 4.06; 95% CI (1.94, 8.47)] were with a higher percentage of IgG positivity compared to rural residents. Anti-rubella IgG positivity was higher in housewives [cOR = 2.94; 95% CI (1.07, 8.04)] compared to self-employed women. Our findings showed a high prevalence of rubella virus exposure, and considerable percentages of recent infection and susceptible women to contracting the infection, emphasizing the importance of congenital rubella syndrome in the research area.

## Introduction

Rubella is a contagious infection caused by the rubella virus that mostly affects children and young adults. Postnatal rubella is commonly acquired through the respiratory route and causes a self-limiting illness, which is mainly characterized by a low-grade fever and skin rash^1^. Infection during pregnancy may result in congenital rubella infection (CRI), with outcomes ranging from subclinical infection, miscarriage, and stillbirth to congenital rubella syndrome (CRS). Defects associated with CRS include blindness, deafness, mental retardation, and congenital heart diseases^2^. The risk of CRS ranges from 10 to 90%, with a higher risk for infection occurring during early gestational age (< 12 weeks), and a lower risk for infection after 20 weeks of gestation^3^.

With the expanded introduction of rubella-containing vaccine (RCV) into countries’ childhood immunization programs, a significant reduction in the burden of infection has been reported, from 670,894 in 2000 to 26,006 in 2018. As of January 2020, 173 (89%) of the 194 World Health Organization (WHO) member countries used rubella immunizations in their national programs^4^; Ethiopia has not yet made the vaccines available. According to estimates, there are 24 and 112 CRS cases per 100,000 live births in urban Addis Ababa and rural Ethiopia, respectively^5^.

In African nations, case-based surveillance for measles has been utilized to identify cases of rubella, with blood samples screened for anti-rubella immunoglobulin (Ig)M, if anti-measles IgM tests were negative^6^. Analysis of such surveillance national data in Ethiopia showed a 12.1% prevalence of anti-rubella IgM from 2004 to 2009^7^ and 15.3% from 2009 to 2015^8^. Case-based surveillance should be supplemented with serosurveys to better understand the epidemiology of rubella infection in a defined population. For instance, despite an increased risk of CRI in Ethiopia, where rubella outbreaks are frequent, there is scarce information available on the infection status of pregnant women^9,10^. Thus, the current study aimed to determine the seroprevalence of rubella virus infection and its distribution by socio-demographic and reproductive characteristics among antenatal clinic clients of Halaba Town public health facilities in southern Ethiopia.

## Methods

### Study design, setting, and period

A facility-based cross-sectional study was conducted in Halaba Town, which is 245 kms south of Addis Ababa and 90 kms from Hawassa City, the capital of the Southern Nations, Nationalities, and Peoples’ Region (SNNPR) and the Sidama Region. The town has five *Kebeles* (the smallest administrative units in Ethiopia) and two public health facilities (one General Hospital and one Health Centre). An estimated 39,507 people live in the town; 51% of which were females, and 23.2% of the females were in the reproductive age group. The town’s population density was four persons per hectare, and the average family size was about six^11^. The study was conducted from March to April 2021.

### Study population

Pregnant women who visited the antenatal clinics at the General Hospital and Health Centre in the town during the study period constituted the study population. Pregnant women older than 18 years were included. However, those who were critically ill at the time of enrolment and refused to participate in the study were excluded.

### Sample size and sampling technique

The sample size was determined using a single population proportion formula (n = z^2^ p (1 − p)/d^2^), assuming an anti-rubella IgG seroprevalence of 86.3% among pregnant women based on a recent report from Hawassa^9^, aiming for a 95% confidence level with 4% precision. Assuming a non-response rate of 5%, the final sample size was estimated to be 299. Consecutive pregnant women were enrolled from the health facilities until the target sample size is obtained.

### Data collection techniques

#### Socio-demographic and reproductive characteristics

Nurses with a 4-years degree gathered data on socio-demographic characteristics (age, residence, marital status, women’s educational and occupational status, and estimated monthly income) as well as reproductive characteristics (gestational age, gravidity, parity, and histories of stillbirth and spontaneous abortion) using pretested and structured questionnaire.

#### Serological analysis

Venous blood samples (about 5 ml) were collected from every study participant, and sera were stored at − 20 °C for a month. Samples were transported to the Public Health Laboratory Institute of SNNPR using a cold box, and stored at − 70 °C until tested. All samples were tested for anti-rubella IgM and IgG using enzyme-linked immunosorbent assay (ELISA) test kits. The Anti-Rubella Virus Glycoprotein (IgM) (EUROIMMUN Medical Laboratory Diagnostics AG, Lubeck, Germany) and the Rubella IgG test kits (DIALAB Diagnostics, Wiener Neudorf, Austria) were used and performed according to the respective manufacturer’s instructions.

### Definitions

Past exposure to rubella virus infection: pregnant women whose blood is tested positive for anti-rubella IgG; thus, protective immunity against the infection.

Recent rubella virus infection: pregnant women who tested positive for IgM antibody.

### Data analysis

Data were double-entered into EpiData version 3.1^12^ and analyzed using SPSS version 20 (IBM Corp., Armonk, NY). Descriptive statistics results were presented using percentages. Binary logistic regression analysis was used to assess the association between socio-demographic and reproductive characteristics and anti-rubella IgM and IgG serostatus. Variables found to have a *p* value < 0.05 were considered significant differences.

### Ethics approval and consent to participate

Ethical approval was obtained from the Institutional Review Board (IRB) (Ref.No: IRB/091/13) of Hawassa University College of Medicine and Health Sciences. Prior to data collection, a supportive letter was obtained from the Halaba Zone Health Department. Study participants were given adequate information regarding the purpose, risk, benefit, and confidentiality of the study. Participation was voluntary, and informed written consent was obtained from each participant. All of the acquired data were linked to code numbers. Positive laboratory results were communicated to the ANC clinics for possible management. All methods were performed in accordance with the relevant guidelines and regulations.

## Results

### Sociodemographic characteristics

Of 308 pregnant women approached during the study period, 9 (2.9%) women were excluded because they refused to participate. Thus, 299 (97.1%) participated in the study. All of the excluded women were from the health centre. The age of participants ranged from 18 to 40 years, with a mean age of 25.4 years (standard deviation, 3.94 years). Half of the participants, 152 (50.8%), were within the age group of 21–30 years, and 210 (70.2%) were urban dwellers. Of the study participants, 131 (43.8%) had no formal education, 247 (82.6%) were housewives, and 118 (39.5%) had an estimated monthly income of ≥ 2001 Ethiopian Birr (Table 1).

**Table 1:** Distribution of anti-rubella IgM and IgG by socio-demographic characteristics of antenatal care clients of Halaba Town public health facilities, southern Ethiopia, 2021.

Variables	Anti-rubella IgM					Anti-rubella IgG			
	No. (%) participants	No. (%) pos	No. (%) neg	cOR (95% CI)	*P* value	No. (%) pos	No. (%) neg	cOR (95% CI)	*P* value
Study facility									
Health centre	217 (72.6)	11 (5.1)	206 (94.9)	1.04 (0.32, 3.37)	0.946	189 (87.1)	28 (12.9)	1	
Hospital	82 (27.4)	4 (4.9)	78 (95.1)	1		76 (92.7)	6 (7.3)	1.88 (0.75, 4.71)	0.180
Age (years)									
≤ 20	45 (15.1)	2 (4.4)	43 (95.6)	1		39 (86.7)	6 (13.3)	1	
21–30	152 (50.8)	7 (4.6)	145 (95.4)	1.04 (0.21, 5.18)	0.964	135 (88.8)	17 (11.2)	1.22 (0.45, 3.31)	0.694
≥ 31	102 (34.1)	6 (5.9)	96 (94.1)	1.34 (0.21, 6.93)	0.724	91 (89.2)	11 (10.8)	1.27 (0.44, 3.69)	0.657
Residence									
Urban	210 (70.2)	11 (5.2)	199 (94.8)	1.18 (0.36, 3.79)	0.788	196 (93.3)	14 (6.7)	4.06 (1.94, 8.47)	< 0.001*
Rural	89 (29.8)	4 (4.5)	85 (95.5)	1		69 (77.5)	20 (22.5)	1	
Educational status									
No formal education	131 (43.8)	7 (5.3)	124 (94.7)	1.64 (0.33, 8.13)	0.546	119 (90.3)	12 (9.2)	1.98 (0.76, 5.20)	0.164
Primary education	60 (20.1)	6 (10.0)	54 (90.0)	3.22 (0.62, 16.7)	0.163	54 (90.0)	6 (10.0)	1.80 (0.58, 5.60)	0.310
Secondary education	60 (20.1)	2 (3.3)	58 (96.7)	1		52 (86.7)	8 (13.3)	1.30 (0.45, 3.76)	0.629
Tertiary education	48 (16.1)	0	48 (100.0)	–		40 (83.3)	8 (16.7)	1	
Occupational status									
Housewife	247 (82.6)	15 (6.1)	232 (93.9)	–		226 (91.5)	21 (8.5)	2.94 (1.07, 8.04)	0.036*
Gov’t employed	28 (9.4)	0	28 (100.0)	–		22 (78.6)	6 (21.4)	0.66 (0.19, 2.34)	0.522
Self-employed	24 (8.0)	0	24 (100.0)	–		17 (70.8)	7 (29.2)	1	
Estimated monthly income									
≤ 1000 birr	81 (27.1)	6 (7.4)	75 (92.6)	4.64 (0.91, 23.6)	0.064	70 (86.4)	11 (13.6)	1	
1001–2000 birr	100 (33.4)	7 (7.0)	93 (93.0)	4.37 (0.89, 21.5)	0.070	88 (88.0)	12 (12.0)	1.15 (0.48, 2.77)	0.751
≥ 2001 birr	118 (39.5)	2 (1.7)	116 (98.3)	1		107 (90.7)	11 (9.3)	1.53 (0.63, 3.72)	0.349

*cOR* crude odds ratio, *CI* confidence interval, *IgM* immunoglobulin M, *IgG* immunoglobulin G, *pos* positive, *neg* negative, *No* number.

*Statistically significant.

### Reproductive health-related characteristics

Of 299 participants, 83 (27.8%) were in their first trimester of pregnancy, 169 (56.5%) were multigravida, and 27 (9.0%) had a history of spontaneous abortion (Table 2).

**Table 2:** Distribution of anti-rubella IgM and IgG by reproductive characteristics of antenatal care clients of Halaba Town public health facilities, southern Ethiopia, 2021.

	Anti-rubella IgM					Anti-rubella IgG			
	No. (%) participants	No. (%) pos	No. (%) neg	cOR (95% CI)	*P* value	No. (%) pos	No. (%) neg	cOR (95% CI)	*P* value
Gravidity									
Primigravida	96 (32.1)	6 (6.2)	90 (93.8)	1		84 (87.5)	12 (12.5)	2.15 (0.80, 5.84)	0.131
Multigravida	169 (56.5)	6 (3.6)	163 (96.4)	0.55 (0.17, 1.76)	0.316	155 (91.7)	14 (8.3)	3.41 (1.30, 8.92)	0.013*
Grand multigravida	34 (11.4)	3 (8.8)	31 (91.2)	1.45 (0.34, 6.16)	0.613	26 (76.5)	8 (23.5)	1	
Parity									
Nulliparous	95 (31.8)	5 (5.3)	90 (94.7)	1		83 (87.4)	12 (12.6)	2.52 (0.69, 9.18)	0.163
Primiparous	68 (22.7)	2 (2.9)	66 (97.1)	0.55 (0.10, 2.90)	0.477	63 (92.6)	5 (7.4)	4.58 (1.06, 19.8)	0.041*
Multiparous	121 (40.5)	5 (4.1)	116 (95.9)	0.776 (0.22, 2.76)	0.695	108 (89.3)	13 (10.7)	3.02 (0.84, 10.9)	0.091
Grand-multiparous	15 (5.0)	3 (20.0)	12 (4.2)	4.50 (0.95, 21.3)	0.058	11 (73.3)	4 (26.7)	1	
Gestational age									
First trimester	83 (27.8)	9 (10.8)	74 (89.2)	4.26 (1.47, 12.4)	0.008*	68 (81.9)	15 (18.1)	1	
Second and third trimesters	216 (72.2)	6 (2.8)	210 (97.2)	1		197 (91.2)	19 (8.8)	2.29 (1.10, 4.75)	0.027*
History of abortion									
Yes	27 (9.0)	1 (3.7)	26 (96.3)	1.4 (0.18, 11.2)	0.744	22 (81.5)	5 (18.5)	1.90 (0.67, 5.41)	0.227
No	272 (91.0)	14 (5.1)	258 (94.9)	1		243 (89.3)	29 (10.7)	1	
History of stillbirth									
Yes	3 (1.0)	1 (–)	2 (–)	10.0 (0.86, 117.8)	0.066	3 (100)	0 (0.0)		
No	296 (99.0)	14 (4.7)	282 (95.3)	1		262 (88.5)	34 (11.5)	–	
History of skin rash									
Yes	6 (2.0)	2 (–)	4 (–)			5 (–)	1 (–)		
No	293 (98.0)	13 (4.4)	280 (95.6)	–		260 (88.7)	33 (11.3)	–	

*cOR* crude odds ratio, *CI* confidence interval, *IgM* immunoglobulin M, *IgG* immunoglobulin G, *pos* positive, *neg* negative, *No* number.

*Statistically significant.

### Seroprevalence of rubella virus infection

Among 299 pregnant women, 15 (5%, 95% CI 2.5–7.5%) were positive for anti-rubella IgM and 265 (88.6%, 95% CI 85.0–92.2%) were positive for anti-rubella IgG. Pregnant women with no IgM or IgG to rubella infection accounted for 11.4%. Of the 299 participants, 11 (3.7%) were found to be positive for both IgM and IgG antibodies.

Tables 1 and 2 show the associations of participants’ socio-demographic and reproductive characteristics with seropositivity for anti-rubella IgM and IgG antibodies. In bivariate logistic regression analyses, pregnant women in their first trimester [crude odds ratio (cOR) = 4.26; 95% CI (1.47, 12.4)] had higher odds of having anti-rubella IgM compared to those in their second and third trimesters taken together. A higher proportion of pregnant women with anti-rubella IgG was found among urban residents [cOR = 4.06; 95% CI (1.94, 8.47)] than rural residents. Compared to self-employed women, housewives were more likely to have anti-rubella IgG positivity (cOR = 2.94; 95% CI (1.07, 8.04)). Further, multigravida women [cOR = 3.41; 95% CI (1.30, 8.92)] had a higher likelihood of having anti-rubella IgG positivity compared to grand multigravida women.

## Discussion

In these serological analyses of rubella infection in pregnant women, we found anti-rubella IgM positivity of 5%, indicating a recent rubella virus infection (or re-infection), and anti-rubella IgG positivity of 88.6%, indicating past exposure to rubella virus infection with development of protective immunity. While a recent rubella infection was more frequently detected among pregnant women in their first trimester, past rubella virus infection was more likely among urban residents, housewives, and multigravida women.

The observed proportion of anti-rubella IgM positivity in this study was comparable with pooled seroprevalence of rubella among pregnant women in sub-Saharan Africa (89.0%) including a single report from southern Ethiopia (86.3%)^9^. Compared to our finding, slightly lower proportions were reported in studies in China (83.3%)^13^ and India (83.4 and 82.3%)^14,15^, as also in northern Ethiopia (79.5%)^10^. Despite the observed variability in the prevalence of rubella infections, studies generally showed high proportions of exposure to rubella infections among pregnant women in various geographical regions where a rubella vaccination has not been introduced or has recently been introduced. Most children aged under 15 years in Africa develop immunity as a result of natural infection^1^. The observed seronegativity of anti-rubella IgG (11.4%) in our study indicates an intermediate level of susceptibility (10–20%) and a medium risk of CRS in the study setting^16^. Our study showed a higher proportion of seronegativity compared to the WHO threshold of < 5% in a childbearing-age woman^17^, which was below the target to be achieved in Africa by 2030^18^.

Our finding of seroprevalence of IgM was higher compared to a result from southern Ethiopia (2.1%)^9^. However, the pooled prevalence of recent rubella infection in sub-Saharan Africa (5.1%) was consistent with our current observation, as also reported recently from Cameroon (5.5% and 5%)^19,20^. On the other hand, the proportion of IgM positivity was lower than that reported in northern Ethiopia (9.5%)^10^ and Nigeria 7.8–38.8%^21–23^. The observed difference in proportions of IgM positivity among the studies might be due to variable endemicity of rubella, testing methods, and population density.

A higher proportion of IgM positivity among pregnant women who were in their first trimester was consistent with studies from Ethiopia^24^ and Tanzania^25^, and it is a major concern in light of an increased risk of developing CRS with decreasing gestational age^3^. Our finding of the higher proportion of anti-rubella IgG positivity among urban dwellers compared to rural ones was consistently reported by several studies in African countries^21,26–28^. The possible reason might be crowded living conditions in urban populations increase the risk of rubella transmission.

Our study has the strength of being one of few such investigations of rubella virus infection among pregnant women in Ethiopia. Findings from this study should inform decision-makers on the prevalence of recent rubella virus infection and the status of susceptibility to rubella virus infection to plan intervention efforts aimed at reducing the incidence of CRS. However, the study faced some limitations. Since participants were recruited from health facilities, findings may not be compared to all pregnant women in the study area. Further, our study might not have been powered adequately to identify socio-demographic and reproductive characteristics associated with rubella infection. The study participants were interviewed in health facilities by health workers, and data collection relied on participants’ self-reports, which might be subject to social desirability, recall, and information bias.

## Conclusion

The high proportion of pregnant women with exposure to the rubella virus, particularly in urban areas, indicates the endemicity of the infection in the Ethiopian context. The observed proportion of recent infection, particularly in pregnant women in their first trimester, may reflect the significance of CRS in the study area. Our findings call for interventions to reduce the risk of CRS, including vaccinating susceptible women of childbearing against the rubella virus and introducing vaccines to the routine childhood immunization programme.

## Data Availability

The datasets generated and/or analysed during the current study are available from the corresponding author, upon reasonable request and with the Institutional Review Board of the Hawassa University College of Medicine and Health Sciences. Restrictions apply to the availability of these clinical data, which caregivers had consented for the collected information to be used for our research study only, and so are not publicly available.
